# Relative Bradycardia and Tachycardia and Their Associations with Adverse Outcomes in Hospitalized COVID-19 Patients

**DOI:** 10.31083/j.rcm2408238

**Published:** 2023-08-18

**Authors:** Petra Bistrovic, Dijana Besic, Tomislav Cikara, Luka Antolkovic, Josip Bakovic, Marija Radic, Josip Stojic, Besa Osmani, Mirna Hrabar, Julija Martinkovic, Diana Delic-Brkljacic, Marko Lucijanic

**Affiliations:** ^1^Cardiology Department, University Hospital Dubrava, 10000 Zagreb, Croatia; ^2^Colorectal Surgery Department, University Hospital Dubrava, 10000 Zagreb, Croatia; ^3^Gastroenterology, Hepatology and Clinical Nutrition Department, University Hospital Dubrava, 10000 Zagreb, Croatia; ^4^Nephrology Department, University Hospital Dubrava, 10000 Zagreb, Croatia; ^5^Endocrinology Department, University Hospital Dubrava, 10000 Zagreb, Croatia; ^6^School of Medicine, University of Zagreb, 10000 Zagreb, Croatia; ^7^Cardiology Department, University Hospital Center Sisters of Mercy, 10000 Zagreb, Croatia; ^8^Hematology Department, University Hospital Dubrava, 10000 Zagreb, Croatia

**Keywords:** COVID-19, bradycardia, tachycardia, fever, arrhythmia, SARS-CoV-2

## Abstract

**Background:**

Relative-tachycardia (RT), a phenomenon of unproportionately high heart-rate elevation in response to fever, has been previously attributed to unfavourable outcomes in severe-inflammatory-response-syndrome (SIRS). Relative heart-rate to body-temperature ratio (RHR) and its prognostic associations in patients with severe and critical coronavirus disease 2019 (COVID-19) have not been investigated.

**Methods:**

We retrospectively analyzed heart-rate and body-temperature data at admission in patients who were hospitalized due to COVID-19 at a tertiary center from March 2020 to June 2021. After excluding patients with known heart rate affecting medications (beta-blockers and other antiarrhythmics) and atrial fibrillation, a total of 3490 patients were analyzed. Patients were divided into quartiles based on RHR on admission, with patients belonging to the 1st quartile designated as having relative-bradycardia (RB) and patients belonging to 4th quartile designated as having RT. Comparisons with baseline clinical characteristics and the course of treatment were done.

**Results:**

There were 57.5% male patients. Median age was 69 years. Most patients had severe or critical COVID-19 at admission. Median heart-rate at the time of hospital admission was 90/min, median body-temperature was 38 °C, and median RHR was 2.36 with interquartile-range 2.07–2.65. RB in comparison to middle-range RHR was significantly associated with older age, higher comorbidity burden, less severe COVID-19 and less pronounced inflammatory profile, and in comparison to RT additionally with higher frequency of hyperlipoproteinemia but lower frequency of obesity. RT in comparison to middle-range RHR was significantly associated with younger age, more severe COVID-19, lower comorbidity burden, lower frequency of arterial hypertension, higher frequency of diabetes mellitus, and more pronounced inflammatory profile. In multivariate analyses adjusted for clinically meaningful parameters, RB patients experienced more favorable survival compared to RT, whereas RT patients experienced higher mortality in comparison to RB and middle-range RHR patients, independently of older age, male sex, higher comorbidity burden and higher COVID-19 severity.

**Conclusions:**

Heart rate and axillary temperature are an indispensable part of a clinical exam, easy to measure, at effectively no cost. RT at admission, as a sign of excessive activation of the sympathetic nervous system, is independently associated with fatal outcomes in COVID-19 patients.

## 1. Introduction

Coronavirus disease 2019 (COVID-19) has been associated with numerous adverse cardiac outcomes due to severe inflammation, endothelial lesions and procoagulant effects [1, 2]. Arrhythmias seem to be the most common cardiac complication of COVID-19 and are commonly observed as a side effect of various treatment options [3, 4, 5, 6]. Arrhythmic phenomena associated with high levels of inflammatory cytokines, subsequent myocardial injury and potential effects of the virus itself on autonomic regulation have been described in COVID-19 [7, 8]. These include relative bradycardia, which has been heterogeneously defined in different works, and relative tachycardia, which has been less well characterized in COVID-19 patients [9, 10, 11, 12]. It is expected for heart rate to rise about 10 beats per minute for every degree in body temperature increase [13]. Among critically ill patients, body temperature is positively correlated with the severity of organ dysfunction [14]. Data observed among septic non-COVID-19 patients suggest that relative tachycardia (4th quartile of relative heart rate (RHR) defined as heart rate divided by body temperature) might be linked to exacerbated sympathetic activation and is associated with a fatal outcome [15]. The COVID-19 pandemic has introduced many markers into clinical practice that were not previously used (e.g., interleukin 6 (IL-6) measurements) and has proved the role of already established methods (e.g., chest ultrasound). However, simple and easily attainable biomarkers have the quality of universal availability and ease of access. Severe and critical COVID has been described as “mirror-like” to sepsis and severe-inflammatory-response-syndrome (SIRS) [16]. Given the similarities of these conditions, we aimed to investigate the effects of increased heart rate temperature ratio in COVID-19 patients.

## 2. Materials and Methods

We retrospectively analyzed heart rate and axillary temperature recorded at admission in patients who were hospitalized due to COVID-19 at our institution from March 2020 to June 2021. Inclusion criteria were being aged 18 or over and a polymerase chain reaction (PCR)-verified COVID-19 infection. Exclusion criteria were a history of atrial fibrillation and the use of beta-blockers and other antiarrhythmic drugs. All patients were Caucasian. The severity of COVID-19 symptoms at the time of hospital admission was classified according to the World Health Organization into mild, moderate, severe and critical. Comorbidities were analyzed both as individual diseases and as cumulative comorbidity burden measured through the Charlson Comorbidity Index (CCI). Laboratory data at admission was also included in the analysis.

To measure RHR, we used the method applied by Leibovici *et al*. [15]. Heartbeats per minute were divided by temperature measured in degrees Celsius. Results for all patients were divided into quartiles. Relative tachycardia was defined as the highest quartile of heart rate-temperature ratio (bpm/°C), whereas relative bradycardia was defined as the lowest quartile.

Statistical methods: The Kolmogorov-Smirnov test was used to assess the normality of distribution for numerical variables. Since results did not follow a normal distribution, they were presented as medians and interquartile ranges (IQR) and were compared between subgroups using the Kruskal-Wallis ANOVA test with a post-hoc test by Conover and Jockheere-Terpstra test for trend. Categorical variables were presented as frequencies and percentages and were compared between groups using the chi-squared test and chi-squared test for trend. Clinical outcomes of interest (in-hospital mortality, high flow oxygen therapy (HFOT), mechanical ventilation (MV), intensive care unit (ICU), bacteremia, arterial thromboses, venous thromboembolism (VTE) and major bleeding), were evaluated during the hospitalization period. Independent associations of RHR with outcomes of interest were evaluated using logistic regression after adjusting for clinically relevant parameters. *p* values < 0.05 were considered to be statistically significant. All analyses were performed using the MedCalc statistical program version 20.109 (MedCalc Software Ltd, Ostend, Belgium).

## 3. Results

### 3.1 Overview of Patient Cohort

A total of 3490 patients with COVID-19 were included in the analysis. There were 2012 (57.5%) male patients and the median age was 69 years. Regarding the intensity of COVID-19 symptoms, 2409 (69%) patients were severely ill and 585 (15%) critically ill at admission. The median Charlson Comorbidity Index was 3. During hospitalization 750 (21.5%) patients required HFOT, 585 (16.8%) required MV, 764 (21.9%) required ICU treatment, and 1010 (28.9%) died. A total of 230 (6.6%) patients experienced VTE, 164 (4.7%) experienced arterial thromboses, 95 (2.7%) experienced major bleeding, and 368 (10.6%) had bacteremia.

### 3.2 Relative Heart Rate and Clinical Associations

**Fig. 1.**
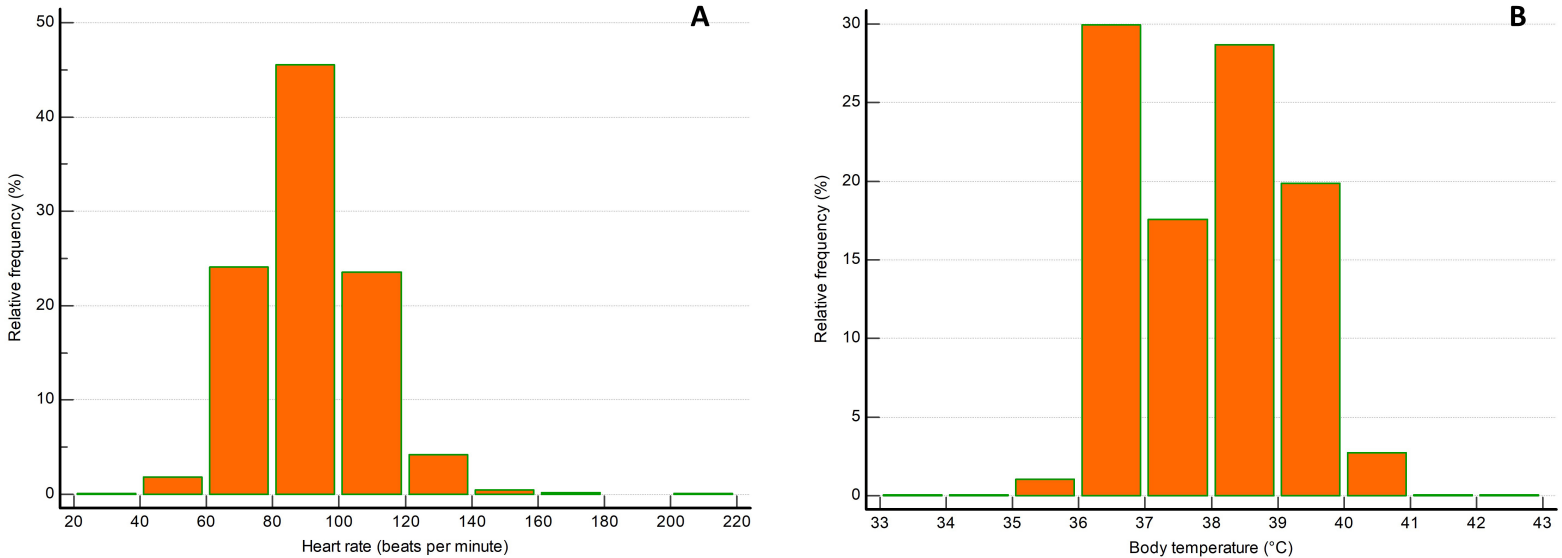
**Distribution of patients regarding (A) heart rate and (B) body temperature at the time of hospital admission**.

Median heart rate at the time of hospital admission was 90/min, IQR (79–100), median body temperature was 38 °C, IQR (36.6–38.8), and median RHR was 2.36, IQR (2.07–2.65). Median heart rate and body temperature across RHR quartiles (from 1st to 4th) were 71/min and 37.9 °C, 84/min and 38 °C, 94/min and 38 °C, and 110/min and 37.7 °C, respectively. Histograms representing heart rate and body temperature distributions are presented in Fig. 1A,B, respectively. Patients’ characteristics in relationship to RHR quartiles are shown in Table 1.

**Table 1.** **Patients’ characteristics stratified according to the relative heart rate (RHR) quartiles**.

		RHR 1st quartile (873)	RHR 2nd quartile (872)	RHR 3rd quartile (873)	RHR 4th quartile (872)	*p* value for difference/trend
Heart rate (beats per minute), median and IQR		71 (65–75)	84 (80–87)	94 (90–98)	110 (104–116)	-
Body temperature (°C), median and IQR		37.9 (36.5–38.9)	38 (36.7–38.9)	38 (36.6–38.7)	37.7 (36.5–38.6)	-
Age (years), median and IQR		70 (60–81)	70 (59–80)	68 (58–78)	66 (53–78)	<0.001* (4 vs 1, 2, 3; 3 vs 1, 2), **
Sex						
	Female	372 (42.6%)	382 (43.8%)	360 (41.2%)	364 (41.7%)	0.716
	Male	501 (57.4%)	480 (56.2%)	513 (58.8%)	508 (58.3%)	
CCI, median and IQR		4 (2–5)	3 (2–5)	3 (2–5)	3 (1–5)	=0.002* (1 vs 2, 3, 4), **
COVID-19 severity						
	Mild	136 (15.6%)	102 (11.7%)	109 (12.5%)	40 (4.6%)	<0.001* (1 vs 2, 4 vs 1, 2, 3), **
	Moderate	44 (5%)	47 (5.4%)	42 (4.8%)	36 (4.1%)	
	Severe	615 (70.4%)	663 (70%)	650 (74.5%)	481 (55.2%)	
	Critical	78 (8.9%)	60 (6.9%)	72 (8.2%)	315 (36.1%)	
ECOG status, median and IQR		2 (1–4)	2 (1–3)	2 (1–3)	2 (1–4)	=0.052
Duration of symptoms (days), median and IQR		6 (1.75–10)	6 (2–10)	6 (1–10)	6 (2–10)	=0.860
Arterial hypertension		488 (55.9%)	504 (57.8%)	462 (52.9%)	449 (51.9%)	=0.046* (4 vs 2, 3 vs 2), **
Diabetes mellitus		171 (19.6%)	193 (22.1%)	222 (25.4%)	219 (25.1%)	=0.011* (4 vs 2, 3 vs 2), **
Hyperlipoproteinemia		146 (16.7%)	125 (14.3%)	124 (14.2%)	112 (12.8%)	=0.027* (4 vs 1), **
Obesity		216 (24.7%)	245 (28.1%)	247 (28.3%)	254 (29.1%)	=0.048* (4 vs 1), **
Prior VTE		39 (4.5%)	24 (2.8%)	26 (3%)	40 (4.6%)	=0.077
Chronic kidney disease		68 (7.8%)	49 (5.6%)	53 (6.1%)	54 (6.2%)	=0.272
Active malignancy		91 (7.4%)	69 (7.9%)	91 (10.4%)	98 (11.2%)	=0.138
Dementia		141 (16.2%)	135 (15.5%)	119 (13.6%)	136 (15.6%)	=0.487
CRP (mg/L), median and IQR		78.20	84.40	93.00	110.20	<0.001* (1 vs 2, 3, 4), **
		(30.73–133.03)	(36.80–147.60)	(41.20–153.35)	(52.75–179.15)	
Ferritin (µg/L), median and IQR		774.00 (415.00–1470.00)	809.00 (408.25–1499.00)	808.00 (410.75–1429.75)	925.00 (487.00–1713.75)	=0.017* (4 vs 1, 2, 3), **
D-dimers (mg/L FEU), median and IQR		1.39 (0.74–2.94)	1.16 (0.65–2.61)	1.15 (0.66–2.82)	1.52 (0.71–3.84)	=0.001* (4 vs 2, 3; 2 vs 1)
WBC (×${\mathrm{10}}^{9}$/L), median and IQR		7.20 (5.50–10.35)	7.50 (5.40–10.70)	7.80 (5.80–11.00)	9.00 (6.5–12.30)	<0.001* (4 vs 1, 2, 3; 3 vs 1), **
Absolute neutrophils (×${\mathrm{10}}^{9}$/L), median and IQR		5.71 (3.91–8.60)	5.88 (4.02–8.60)	6.20 (4.20–9.13)	7.39 (5.04–10.73)	<0.001* (4 vs 1, 2, 3; 3 vs 1), **
Absolute lymphocytes (×${\mathrm{10}}^{9}$/L), median and IQR		0.83 (0.55–1.20)	0.85 (0.60–1.23)	0.81 (0.59–1.20)	0.81 (0.55–1.23)	=0.697
Hemoglobin (g/L), median and IQR		128.00 (115.00–141.00)	130.00 (118.00–141.00)	130.00 (115.00–142.00)	131 (115.00–143.00)	=0.156**
Platelets (×${\mathrm{10}}^{9}$/L), median and IQR		220.00 (164.00–287.50)	227.00 (166.00–301.75)	238.00 (179.00–309.00)	242.00 (181.00–316.75)	<0.001* (4 vs 1, 2; 3 vs 1, 2), **
IL-6 (pg/mL), median and IQR		35.00 (11.13–107.26)	38.66 (12.95–89.72)	36.47 (13.80–103.79)	51.69 (20.94–115.71)	=0.182**
Procalcitonin (ng/mL), median and IQR		0.17 (0.08–0.47)	0.15 (0.08–0.45)	0.19 (0.09–0.56)	0.30 (0.10–1.26)	<0.001* (4 vs 1, 2, 3; 3 vs 2), **

Abbreviations: RHR, relative heart rate; IQR, interquartile range; COVID-19, coronavirus disease 2019; VTE, venous thromboembolism; CCI, Charlson Comorbidity Index; ECOG, Eastern Cooperative Oncology Group; CRP, C reactive protein; WBC, white blood cell count; IL-6, interleukin 6. *statistically significant difference at level *p* < 0.05, ** statistically significant trend at level *p* < 0.05.

Relative bradycardia (1st quartile) in comparison to middle-range RHR (2nd and 3rd quartiles) was significantly associated with older age, higher comorbidity burden, less severe COVID-19 at admission, lower C reactive protein (CRP), higher D-dimers, reduced white blood cell count (WBC), and reduced platelets (*p* < 0.05 for all analyses). Relative bradycardia (1st quartile) in comparison to relative tachycardia was similarly associated with older age, less severe COVID-19 at admission, higher comorbidity burden, higher frequency of hyperlipoproteinemia but lower frequency of obesity, lower CRP, lower ferritin, reduced WBC, reduced platelets and lower procalcitonin (*p* < 0.05 for all analyses). Relative tachycardia (4th quartile) in comparison to middle-range RHR (2nd and 3rd quartiles) was significantly associated with younger age, more severe COVID-19 at admission, lower comorbidity burden, lower frequency of arterial hypertension, higher frequency of diabetes mellitus, higher ferritin, higher D-dimers, increased WBC, increased platelets and higher procalcitonin (*p* < 0.05 for all analyses). In addition, statistically significant trends of increase in COVID-19 severity, frequencies of diabetes mellitus and obesity, CRP, ferritin, WBC, hemoglobin, platelets, IL-6 and procalcitonin, as well as statistically significant trends of decrease in frequencies of age, comorbidity burden, frequencies of arterial hypertension and hyperlipoproteinemia were observed over rising quartiles of RHR (*p* < 0.05 for all analyses). No significant relationships of RHR with sex, functional status at admission, duration of symptoms or other comorbidities (prior venous thromboembolisms, chronic kidney disease, malignant disease or dementia) were recognized.

### 3.3 Associations of Relative Heart Rate with Clinical Outcomes

**Fig. 2.**
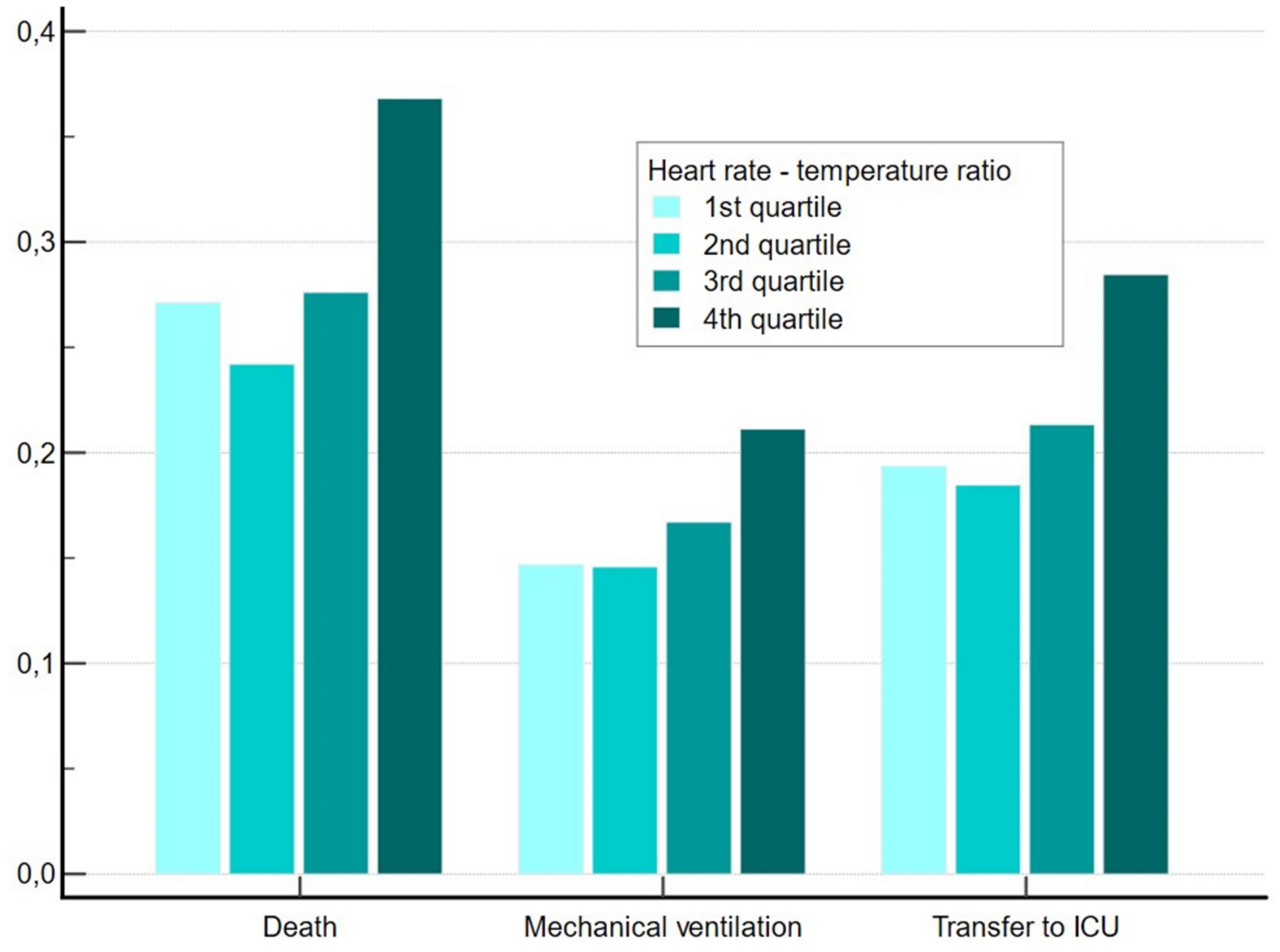
**Associations of relative heart rate quartiles with clinical outcomes of in-hospital mortality, mechanical ventilation and intensive care unit (ICU) use**.

Table 2 presents univariate associations between RHR quartiles and clinical outcomes. In univariate analyses, patients in lower RHR quartiles had a lower likelihood of death during hospitalization (27.1%, 24.4%, 27.6%, 36.8%, *p* < 0.05 both for difference between quartiles and for trend), were less likely to require mechanical ventilation (14.7%, 14.6%, 16.7%, 21.1%, *p* < 0.05 both for difference between quartiles and for trend) and less likely to be transferred to ICU (19.4%, 18.5%, 21.3%, 28.4%, *p* < 0.05 both for difference between quartiles and for trend), as shown in Fig. 2. Patients belonging to lower RHR quartiles were also less likely to require HFOT support (18.9%, 19.5%, 21.8%, 25.8%, *p* < 0.05 both for difference between quartiles and for trend), to experience bacteriemia (10.2%, 8.5%, 9.7%, 13.8%, *p* < 0.05 both for difference between quartiles and for trend), to experience VTE (4.8%, 5.4%, 7.6%, 8.6%, *p* < 0.05 both for difference between quartiles and for trend) and to experience major bleeding (2.1%, 2.4%, 2.5%, 3.9%, *p* < 0.05 for trend).

**Table 2.** **Univariate associations of relative heart rate stratified at quartiles with clinical outcomes during hospitalization**.

	RHR 1st quartile (873)	RHR 2nd quartile (873)	RHR 3rd quartile (872)	RHR 4th quartile (872)	*p* value for difference/trend
Duration of hospitalization (days), median and IQR	10 (6–15)	10 (6–16)	10 (6–16)	9 (6–15)	=0.217
Death during hospitalization	237 (27.1%)	211 (24.2%)	241 (27.6%)	321 (36.8%)	<0.001* (4 vs 1, 2, 3), **
HFOT	165 (18.9%)	170 (19.5%)	190 (21.8%)	225 (25.8%)	=0.002* (4 vs 1, 2, 3), **
MV	128 (14.7%)	127 (14.6%)	146 (16.7%)	184 (21.1%)	<0.001* (4 vs 1, 2, 3), **
ICU	169 (19.4%)	161 (18.5%)	186 (21.3%)	248 (28.4%)	<0.001* (4 vs 1, 2, 3), **
Bacteriaemia	89 (10.2%)	74 (8.5%)	85 (9.7%)	120 (13.8%)	=0.003* (4 vs 1, 2, 3), **
Arterial thrombosis	51 (5.8%)	38 (4.4%)	41 (4.7%)	34 (3.9%)	=0.259
VTE	42 (4.8%)	47 (5.4%)	66 (7.6%)	75 (8.6%)	=0.004* (4 vs 1, 2; 3 vs 1), **
Major bleeding	18 (2.1%)	21 (2.4%)	22 (2.5%)	34 (3.9%)	=0.091**

*statistically significant difference at level *p* < 0.05, ** statistically significant trend at level *p* < 0.05. Abbreviations: IQR, interquartile range; RHR, relative heart rate; HFOT, high flow oxygen therapy; MV, mechanical ventilation; ICU, intensive care unit; VTE, venous thromboembolism.

We further analyzed the results using the multivariate logistic regression models adjusted for clinically meaningful parameters. The results are shown in Table 3, RHR associated risks are presented twice for the same models (first using relative bradycardia — 1st quartile and second using relative tachycardia — 4th quartile as a reference category). Being in the 1st quartile was significantly associated with a lower risk of death in comparison to 4th quartile, and belonging to the 4th quartile was significantly associated with higher risk of death in comparison to the 1st, 2nd and 3rd quartiles, independently of older age, male sex, higher Charlson Comorbidity Index and higher COVID-19 severity. Being in the 1st quartile was significantly associated with lower risk of MV in comparison to the 4th quartile, and belonging to the 4th quartile was significantly associated with a higher risk of MV in comparison to the 1st and 2nd quartiles, independently of male sex. Being in the 1st quartile was significantly associated with a lower VTE risk in comparison to the 3rd and 4th quartiles, and belonging to the 4th quartile was significantly associated with a higher VTE risk in comparison to the 1st and 2nd quartiles, independently of higher COVID-19 severity. Belonging to the 1st quartile was significantly associated with lower major bleeding risk in comparison to the 4th quartile (and oppositely), independently of the higher Charlson Comorbidity Index. Belonging to the 4th quartile was significantly associated with a higher risk of bacteremia in comparison to the 2nd and 3rd quartiles, independently of male sex and higher COVID-19 severity. No significant association could be established between relative bradycardia (1st quartile) and the risk of arterial thromboses and bacteremia, and between relative tachycardia (4th quartile) and the risk of arterial thromboses.

**Table 3.** **Multivariate logistic regression models assessing associations of relative heart rate stratified at quartiles with outcomes during hospitalization**.

Outcome		In-hospital mortality	Mechanical ventilation	VTE	Arterial thrombosis	Major bleed	Bacteriaemia
RHR							
	1st RHR quartile	Reference value	Reference value	Reference value	Reference value	Reference value	Reference value
	2nd RHR quartile	*p* = 0.267	*p* = 0.731	*p* = 0.716	*p* = 0.215	*p* = 0.619	*p* = 0.631
		OR 0.87 (0.69 to 1.11)	OR 0.95 (0.73 to 1.25)	OR 1.08 (0.71 to 1.66)	OR 0.76 (0.49 to 1.17)	OR 1.18 (0.62 to 2.22)	OR 0.79 (0.57 to 1.10)
	3rd RHR quartile	*p* = 0.223	*p* = 0.387	*p* = 0.028*	*p* = 0.443	*p* = 0.611	*p* = 0.516
		OR 1.16 (0.91 to 1.47)	OR 1.12 (0.86 to 1.47)	OR 1.57 (1.05 to 2.34)	OR 0.85 (0.55 to 1.30)	OR 1.18 (0.62 to 2.23)	OR 0.90 (0.66 to 1.24)
	4th RHR quartile	*p * < 0.001*	*p* = 0.030*	*p* = 0.010*	*p* = 0.181	*p* = 0.042*	*p* = 0.185
		OR 1.86 (1.47 to 2.35)	OR 1.33 (1.03 to 1.72)	OR 1.68 (1.13 to 2.450)	OR 0.74 (0.47 to 1.16)	OR 1.84 (1.02 to 3.31)	OR 1.22 (0.91 to 1.65)
RHR							
	4th RHR quartile	Reference value	Reference value	Reference value	Reference value	Reference value	Reference value
	3rd RHR quartile	*p * < 0.001*	*p* = 0.185	*p* = 0.696	*p* = 0.554	*p* = 0.118	*p* = 0.045*
		OR 0.62 (0.50 to 0.78)	OR 0.85 (0.66 to 1.08)	OR 0.93 (0.66 to 1.32)	OR 1.15 (0.72 to 1.84)	OR 0.64 (0.37 to 1.12)	OR 0.74 (0.55 to 0.99)
	2nd RHR quartile	*p * < 0.001*	*p* = 0.010*	*p* = 0.020*	*p* = 0.889	*p* = 0.117	*p* = 0.006*
		OR 0.47 (0.37 to 0.60)	OR 0.72 (0.56 to 0.93)	OR 0.64 (0.44 to 0.94)	OR 1.03 (0.64 to 1.67)	OR 0.64 (0.37 to 1.12)	OR 0.65 (0.48 to 0.88)
	1st RHR quartile	*p * < 0.001*	*p* = 0.03*	*p* = 0.010*	*p* = 0.181	*p* = 0.042*	*p* = 0.185
		OR 0.54 (0.43 to 0.68)	OR 0.75 (0.58 to 0.97)	OR 0.59 (0.40 to 0.88)	OR 1.36 (0.87 to 2.14)	OR 0.54 (0.30 to 0.98)	OR 0.82 (0.61 to 1.10)
Age		*p * < 0.001*	*p* = 0.677	*p* = 0.668	*p* = 0.345	*p* = 0.495	*p* = 0.135
		OR 1.04 (1.03 to 1.05)	OR 1.0 (1.0 to 1.01)	OR 1.00 (1.00 to 1.01)	OR 1.01 (0.99 to 1.02)	OR 0.99 (0.98 to 1.01)	OR 0.99 (0.98 to 1.00)
Male sex		*p * < 0.001*	*p * < 0.001*	*p* = 0.173	*p* = 0.033*	*p* = 0.561	*p* = 0.002*
		OR 1.36 (1.14 to 1.61)	OR 1.44 (1.19 to 1.74)	OR 0.83 (0.63 to 1.09)	OR 1.44 (1.03 to 2.00)	OR 1.14 (0.74 to 1.74)	OR 1.43 (1.14 to 1.81)
Charlson Comorbidity Index		*p * < 0.001*	*p* = 0.207	*p* = 0.421	*p * < 0.001*	*p* = 0.05*	*p* = 0.072
		OR 1.24 (1.19 to 1.29)	OR 1.03 (0.98 to 1.08)	OR 0.97 (0.90 to 1.05)	OR 1.12 (1.05 to 1.21)	OR 1.10 (1.10 to 1.21)	OR 1.05 (1.0 to 1.11)
Severe or critical COVID-19		*p * < 0.001*	*p* = 0.998	*p * < 0.001*	*p* = 0.005*	*p* = 0.181	*p * < 0.001*
		OR 28.81 (14.68 to 56.55)	OR -	OR 2.38 (1.43 to 3.98)	OR 0.56 (0.38 to 0.84)	OR 1.63 (0.80 to 3.31)	OR 1.05 (1.0 to 1.11)

*statistically significant at level *p* < 0.05. Odds ratios with 95% confidence intervals are shown. Abbreviations: RHR, relative heart rate; VTE, venous thromboembolism; OR, odds ratio; COVID-19, coronavirus disease 2019.

## 4. Discussion

Our study is the first to report on the outcomes of patients with COVID-19 and relative bradycardia/tachycardia. An association between relative tachycardia and unfavorable clinical outcomes was first described in patients with SIRS and sepsis by Leibovici* et al*. [15] in 2007. Patients who had tachycardia which was disproportionate to their grade of fever at admission had increased 30-day mortality, independently of other factors typically associated with fatal outcomes. So far, no published data on this phenomenon in COVID-19 patients exist.

Fever at admission, as well as prolonged fever, have been associated with negative outcomes and death in patients with COVID-19 [17, 18]. However, the authors researching relative tachycardia in sepsis patients have attributed negative effects of RHR mostly to tachycardia, caused by overstimulation of the sympathetic nervous system due to SIRS/sepsis [15]. Catecholamine release caused by inflammation inflicts a stressful reaction, which in turn potentiates sympathetic stimulation resulting in tachycardia, as well as other systemic adrenergic effects [19]. Tachycardia is known to worsen outcomes in critically ill patients given the increased myocardial oxygen requirements and shortening of diastole resulting in a decrease in myocardial perfusion and further ischemia [20]. Studies of beta-blockers in sepsis have shown their potential benefit attributed to the adrenergic blockade, impacting cardiac, immunological, metabolic and coagulative function [21].

COVID-19, given the dynamic in proinflammatory cytokines and coagulopathy, has shown similarities to sepsis, especially during the chronic inflammation phase after its initial acute infective phase [22]. Hypoxia, an imbalance of angiotensin-converting enzyme (ACE)-1 and ACE-2, immunological factors and emotional stress caused by COVID-19 exacerbate SNS activation [23]. SARS-CoV-2 virus and the cytokines released during a cytokine storm have been shown to cause neuroinflammation further aggravating the sympathetic response [16]. Drawing a parallel to SIRS and sepsis, a pilot study conducted on critically ill COVID-19 patients treated with metoprolol has shown a positive impact of beta-blocker therapy in terms of decreased local inflammation in the lungs and shortened ICU stay, however, the impact of metoprolol on fatal outcome was not reported [24]. Other treatment options affecting heart rate have also shown beneficial results. Sinus bradycardia has been described as the most common cardiovascular effect of remdesivir, the first approved antiviral drug for the treatment of COVID-19 [5]. Although initially considered a side effect, further research has demonstrated decreased mortality in patients who developed bradycardia during remdesivir treatment [25]. Similarly, patients with conditions that might benefit from heart rate control like atrial fibrillation might have reduced mortality when treated with remdesivir [26].

Our results show that relative tachycardia in patients with COVID-19 is a phenomenon associated with increased mortality compared to patients with relative bradycardia, as well as to patients with middle-range RHR, regardless of other factors contributing to fatal outcomes. Compared to previously published research which associated relative bradycardia (defined differently than in our study) with increased mortality [11, 12], there were no statistical differences in unfavorable outcomes for patients with relative bradycardia. On the contrary, our data suggest that relative bradycardia seems to be protective compared to relative tachycardia regarding risks of mortality, mechanical ventilation, VTE and major bleeding, whereas lower VTE rates were observed in comparison to middle-range RHR.

Further research is necessary to establish whether early therapeutic intervention can affect the outcomes. Aside from standard antipyretic therapy, the use of beta-blockers — particularly a short course of metoprolol, has shown potentially beneficial results, with a low risk of complications. However, studies on larger sample sizes in different COVID severity groups are necessary. Nevertheless, heart rate and axillary temperature are an indispensable part of a clinical exam, easy to measure, at effectively no cost. This makes relative tachycardia a potential prognostic factor for patients with COVID-19 that can be easily utilized. Identifying preventative measures and early treatment could potentially prevent further disease progression and complications, therefore increasing survival. Further research is also needed to validate defined cut-off values for the heart rate-temperature ratio.

Limitations of our study include it being single-center, retrospective research, lack of data on pharmacotherapy received during hospitalization and its potential impact on the outcome. Symptom severity, symptom onset date, and specific treatments prior to hospitalization have been obtained from the medical records and could not be additionally verified. Conditions like inappropriate sinus tachycardia, an uncommon disorder that affects patients with no evident cardiac disease, as well as postural orthostatic tachycardia syndrome (POTS), a complex multisystem disorder characterized by orthostatic intolerance and tachycardia that may be triggered by viral infections, could not be excluded with certainty. Although no sex-related differences were observed between RHR quartiles, the presence of sex bias/sex paradox in retrospective COVID-19 studies should be considered, i.e., differences in demographic and comorbidity background of male and female populations and their interactions with sex in relationship to clinical outcomes [27, 28, 29]. It remains a question whether RHR should be additionally adjusted for parameters such as age, sex, body mass index, hypoxia, etc. to provide more patient-specific measures. However, this might result in a more cumbersome calculation and diminish its clinical usefulness as a rapidly attainable and simple parameter. The main strengths of our work are a large study sample and a real-life patient cohort representative of elderly patients with comorbidities with mostly severe or critical COVID-19 treated in a tertiary referral center.

## 5. Conclusions

Heart rate and axillary temperature are an indispensable part of a clinical exam, easy to measure, at effectively no cost. Comparable to SIRS and sepsis, RT at admission, as a sign of excessive activation of the sympathetic nervous system, is independently associated with fatal outcomes in COVID-19 patients.

## Data Availability

Data are available from the corresponding author upon reasonable request.
